# Characterization of interfacial socket pressure in transhumeral prostheses: A case series

**DOI:** 10.1371/journal.pone.0178517

**Published:** 2017-06-02

**Authors:** Jonathon S. Schofield, Katherine R. Schoepp, Heather E. Williams, Jason P. Carey, Paul D. Marasco, Jacqueline S. Hebert

**Affiliations:** 1 Faculty of Engineering, Department of Mechanical Engineering, University of Alberta, Edmonton, Alberta, Canada; 2 Faculty of Medicine & Dentistry, Division of Physical Medicine & Rehabilitation, University of Alberta, Edmonton, Alberta, Canada; 3 Lerner Research Institute, Department of Biomedical Engineering, Cleveland Clinic, Cleveland, Ohio, United States of America; 4 Advanced Platform Technology Center of Excellence, Louis Stokes Cleveland Department of Veterans Affairs Medical Center, Cleveland, Ohio, United States of America; 5 Glenrose Rehabilitation Hospital, Alberta Health Services, Edmonton, Alberta, Canada; Northwestern University, UNITED STATES

## Abstract

One of the most important factors in successful upper limb prostheses is the socket design. Sockets must be individually fabricated to arrive at a geometry that suits the user’s morphology and appropriately distributes the pressures associated with prosthetic use across the residual limb. In higher levels of amputation, such as transhumeral, this challenge is amplified as prosthetic weight and the physical demands placed on the residual limb are heightened. Yet, in the upper limb, socket fabrication is largely driven by heuristic practices. An analytical understanding of the interactions between the socket and residual limb is absent in literature. This work describes techniques, adapted from lower limb prosthetic research, to empirically characterize the pressure distribution occurring between the residual limb and well-fit transhumeral prosthetic sockets. A case series analyzing the result of four participants with transhumeral amputation is presented. A Tekscan VersaTek pressure measurement system and FaroArm Edge coordinate measurement machine were employed to capture socket-residual limb interface pressures and geometrically register these values to the anatomy of participants. Participants performed two static poses with their prosthesis under two separate loading conditions. Surface pressure maps were constructed from the data, highlighting pressure distribution patterns, anatomical locations bearing maximum pressure, and the relative pressure magnitudes. Pressure distribution patterns demonstrated unique characteristics across the four participants that could be traced to individual socket design considerations. This work presents a technique that implements commercially available tools to quantitatively characterize upper limb socket-residual limb interactions. This is a fundamental first step toward improved socket designs developed through informed, analytically-based design tools.

## 1. Introduction

One of the most crucial factors for the successful use of an upper limb (UL) prosthesis is the design of the prosthetic socket[1]. The socket encompasses the user’s residual limb (RL) and functions as the point of attachment securing the prosthetic components to the user. It is at this crucial junction where the soft tissue of the user’s RL must interface with the rigid materials of the prosthesis. Consequently, a prosthetic socket must be custom-designed to accommodate the individual’s morphology, achieve suspension of the prosthesis, and aid in control by securely and efficiently transmitting intended movements. This not only promotes the user’s ability to move and manipulate their prosthesis, but in a system that is otherwise absent of direct sensory feedback, may help promote indirect feedback such as position of the prosthetic device. The term ‘socket fit’ broadly describes the quantitative and qualitative factors impacting prosthetic comfort, suspension and stability on the RL. Both fit and the corresponding comfort have substantial implications on user satisfaction; how long (or if) the user will tolerate wearing their prosthesis; and, ultimately, the success of an UL prosthetic prescription[2–5]. Clinically, the implications of fit are well acknowledged and much of a prosthetist’s effort is specifically dedicated to the design and fabrication of the socket[6].

The primary challenge in socket design and fabrication is achieving socket geometry that appropriately distributes pressure across the RL. The socket must couple the prosthesis to the user’s underlying bony structure through deliberate compression and relief in appropriate areas of the surrounding soft tissues. As a physical consequence, some skeletal motion will inherently be lost due to the deformable nature of residual tissues and not fully translated to movement of the prosthesis[7]. This decreases the stability of the system and may result in a reduced range of motion and inappropriate loading of the residual anatomy[7]. Therefore, a technical design challenge exists: mechanical stability will increase with tissue compression and relief at physiologically and mechanically appropriate locations on the residual limb. The resulting contact pressures magnitudes and locations must therefore be strategically considered during socket fabrication to reduce the risk of discomfort, tissue irritation, and damage. This challenge becomes more relevant for those with more proximal amputations, as additional prosthetic components (elbows and shoulders) are required. This increases the weight and reduces the users control over their device; ultimately amplifying the demand placed on the socket and the user’s RL.

Adding to these challenges, Lake et al., describe a paradox which they refer to as “the upper extremity dilemma”[8]. The prevalence of lower limb amputations far outweighs that of UL[9]. As the field of UL prosthetics becomes increasingly technical and specialized, it becomes challenging for many prosthetists to expand their UL knowledge as significantly fewer UL clients are seen[10]. This results in a group of affected individuals requiring highly specialized expertise from clinicians who have often limited practice in addressing their needs. This is further magnified for more proximal levels of UL amputation, such as transhumeral, as the demand on the prosthetic socket increases and prevalence further decreases[9]. When compared to those with more distal amputations, prosthetic users with transhumeral or shoulder level amputations are more likely to be dissatisfied and reject their prosthesis[3]. Underlying reasons may include dissatisfaction with prosthetic functionality or appearance, however a chief contributor lays in prosthetic comfort [5,11] specifically at the socket interface[5].

In practice, socket fabrication relies heavily on experienced-based, heuristic techniques. Although literature does report novel socket designs for improved suspension and function[7,8] very little analytical documentation and quantitative descriptions of UL socket-RL interactions have been published. This limits the understanding of the implications of socket design decisions. Dally et al. performed work with a group of participants with upper limb amputation (3 transradial, 3 transhumeral and 3 shoulder disarticulation participants)[12]. They used a Tekscan sensor system for the measurement of contact pressure between the RL and prosthesis to investigate the relationship between maximum pressures and discomfort. They found a combination of weak and strong correlations and concluded that this relationship is variable and patient specific. However this study reported maximum pressures exclusively and did not report the anatomical locations at which these pressures occurred, limiting the translation of this work to aid informed socket design decisions.

In lower limb prostheses, measurements and mapping pressures to participants’ anatomy are commonly performed using commercially available systems such as the Tekscan F-Socket or VersaTek[13,14]. The accuracy and repeatability of such systems have been well documented[15–17] and it has been suggested they are adequate in indicating areas of high pressure at the socket-RL interface[16]. Pressure measurement and mapping of lower-limb prosthetic sockets has improved the understanding of prosthetic fit at a very fundamental level and has helped facilitated objectively based socket designs[18–21]. Yet these techniques do not translate directly to the UL. There is much complexity in the diverse set of movements performed with UL prostheses relative to the cyclical, high pressure loading patterns experienced in the lower limb. Therefore, applying pressure measurement and mapping to UL sockets holds the potential to characterize these unique physical differences and develop foundational knowledge to aid in socket design and fabrication processes.

This work describes empirical techniques enabling the analytical characterization of socket-RL interface pressures. We adapt lower-limb socket measurement methods to describe pressure distributions across the RL in terms of distribution patterns, load bearing anatomy, and relative magnitude of interfacial pressures. These techniques were employed to test four transhumeral participant wearing well-fit prosthetic sockets.

## 2. Methods

**Fig 1:**
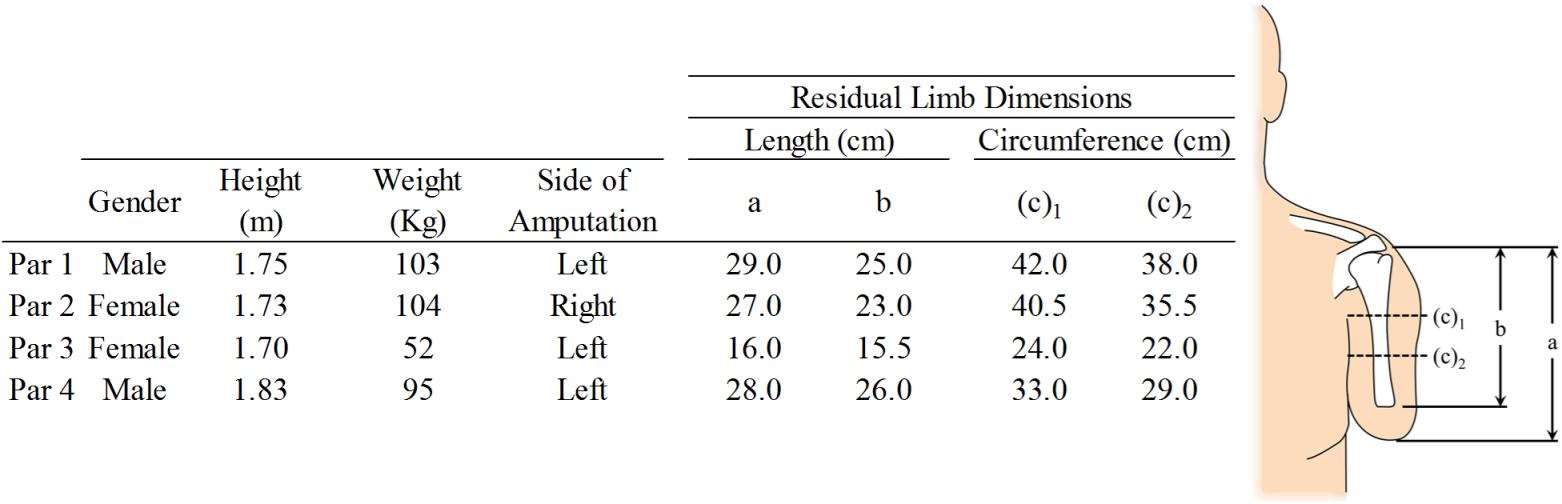
Participant demographics and residual limb characteristics, par represents the participant number, (c)_1_ and (c)_2_ denote circumference measurements taken at the axilla and mid-length of the limb respectively, a denotes the residual limb length taken from the acromion to distal tip of the limb, and b represents the limb length from the acromion to the distal residual humeral tip.

Four participants with transhumeral amputation were recruited. Participant demographics and RL geometries are reported in Fig 1. All participants wore a body-powered prosthesis with voluntary opening terminal device and prosthetic liner. A description of each participant’s prosthetic components is included in Table 1. Ethics approval was obtained through the University of Alberta’s institutional review board and participants provided informed consent prior to participation.

**Table 1:** Descriptions of participants’ prosthetic components.

	Terminal Device	Elbow	Liner	Harness
Participant 1	Hosmer 555 Lyre Hook	Ottbock ErgoArm	WillowWood Alpha Medium	Figure-eight (bilaterally connected control cable)
Participant 2	Ottobock 8K23 Hand	Ottbock ErgoArm	WillowWood Alpha Medium	Figure-eight
Participant 3	Hosmer 555 Lyre Hook	Ottbock ErgoArm	Ossur Iceross Upper-X	Figure-eight
Participant 4	Hosmer 555 Lyre Hook	Ottbock ErgoArm	WillowWood Alpha Medium	Figure-eight

### 2.1 Socket fit

Participants were recruited through the Glenrose Rehabilitation Hospital’s prosthetics department. Subjects were enrolled in the study within approximately 60 days following the delivery of a newly refit or adjusted prosthetic socket. Each participant’s socket was evaluated by a certified prosthetist, and based on their clinical expertise, deemed a ‘well-fit’ socket prior to testing. To confirm the quality of socket fit, each participant completed an OPUS Satisfaction with Device survey modified to present questions relevant to prosthetic socket fit[22]. This survey and results are included in the Supplementary Material (S1 Table).

### 2.2 Socket pressure measurements

A Tekscan VersaTek system with 9811E sensors (Tekscan Inc., Boston, USA) was used to capture contact pressures acting on the RL within the prosthetic socket. Each sensor contained 96 sensels; therefore, being capable of capturing 96 simultaneous discrete readings. This system was selected as the thin flexible profile of the sensors permits socket-RL interface measurements without the need to modify the socket. Sensors were adhered directly to each participants RL using double sided adhesive tape. One or two sensors, which were trimmed as necessary and positioned to maximize coverage over the residual limb, were used for each participant.

**Fig 2:**
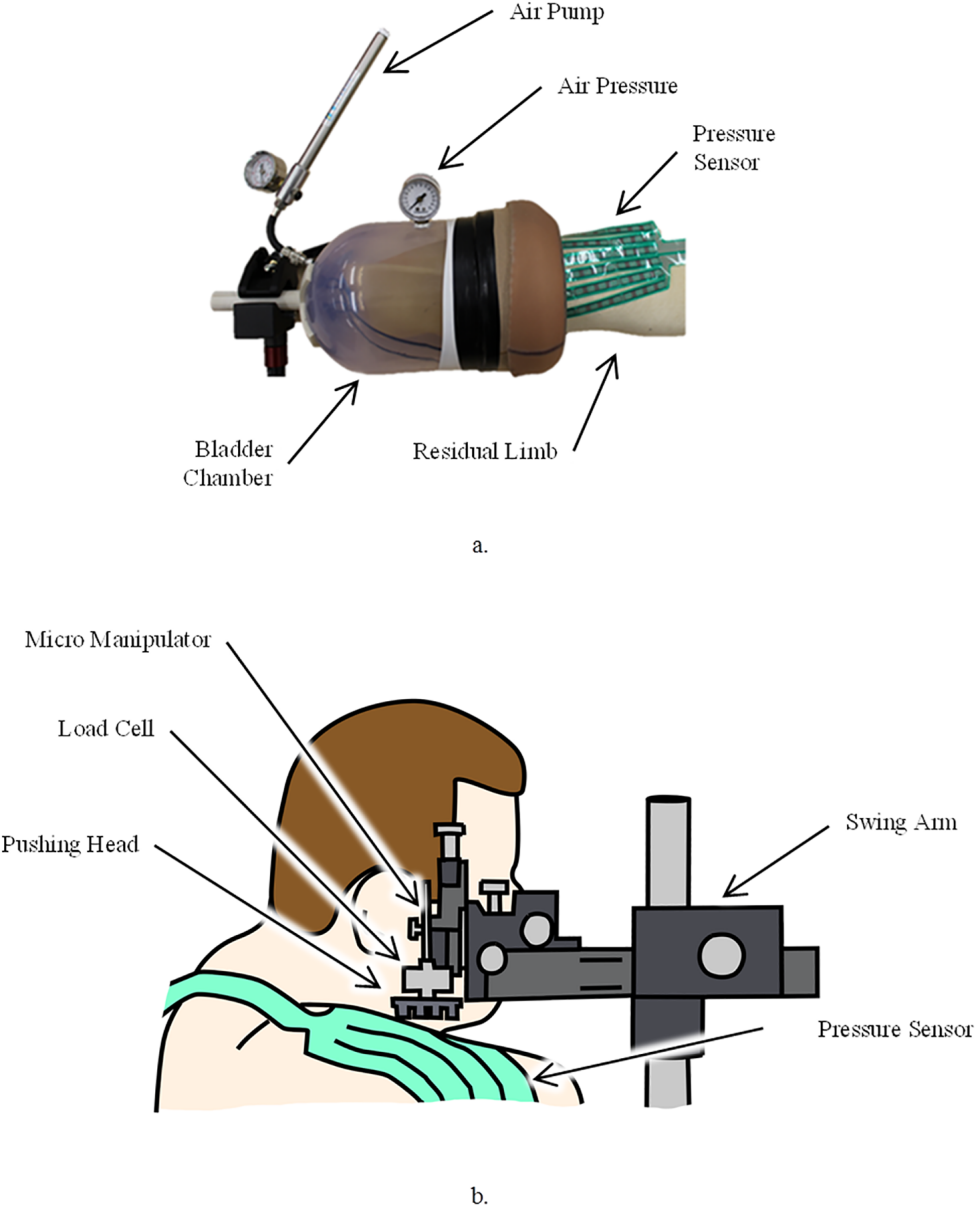
Equilibration and calibration apparatuses, a. equilibration setup schematically described and demonstrated on a participant, b. calibration setup demonstrated on a participant.

As required by the Tekscan VersaTek software, each sensor underwent an equilibration and calibration procedure. Typically these activities would be performed with the sensor on a flat, rigid surface at room temperature; however literature suggests improved accuracy with thin film-sensors if calibration occurs in an environment as close to their intended use as possible[23]. Therefore, custom apparatuses were fabricated to allow for these activities to be performed while the sensors were adhered to the participant’s residual limb. The equilibration procedure required equal pressure to be distributed across the whole surface area of the sensor. This was achieved using an inflatable bladder system whereby the participant placed their RL into a chamber where a bladder is inflated around the RL (Fig 2A). Two equilibration points were captured in the software, baseline atmospheric and 10 kPa. Calibration was performed using a load cell affixed to a custom pushing head, and an apparatus allowing for the application of known loads to the sensors on the RL (Fig 2B). A two point calibration was employed in the Tekscan software which captured the sensor response to a baseline 0 Newton force and 10 Newton force.

The location of each sensor relative to the anatomy of the participants was captured using a FaroArm Edge Coordinate Measurement Machine (FaroArm, Coventry, UK). Each participant’s RL was supported by a stationary arm rest to prevent movement of the limb during this process. The three dimensional position of each sensor’s sensels relative to a predefined coordinate system were logged. Additionally the coordinates of five anatomical landmarks were captured to geometrically register the relative position of each sensor to the participant’s anatomy (acromion, lateral distal point of the residual humerus, mid-bicep muscle belly, mid-triceps muscle belly and most distal tip of the residual limb).

**Fig 3:**
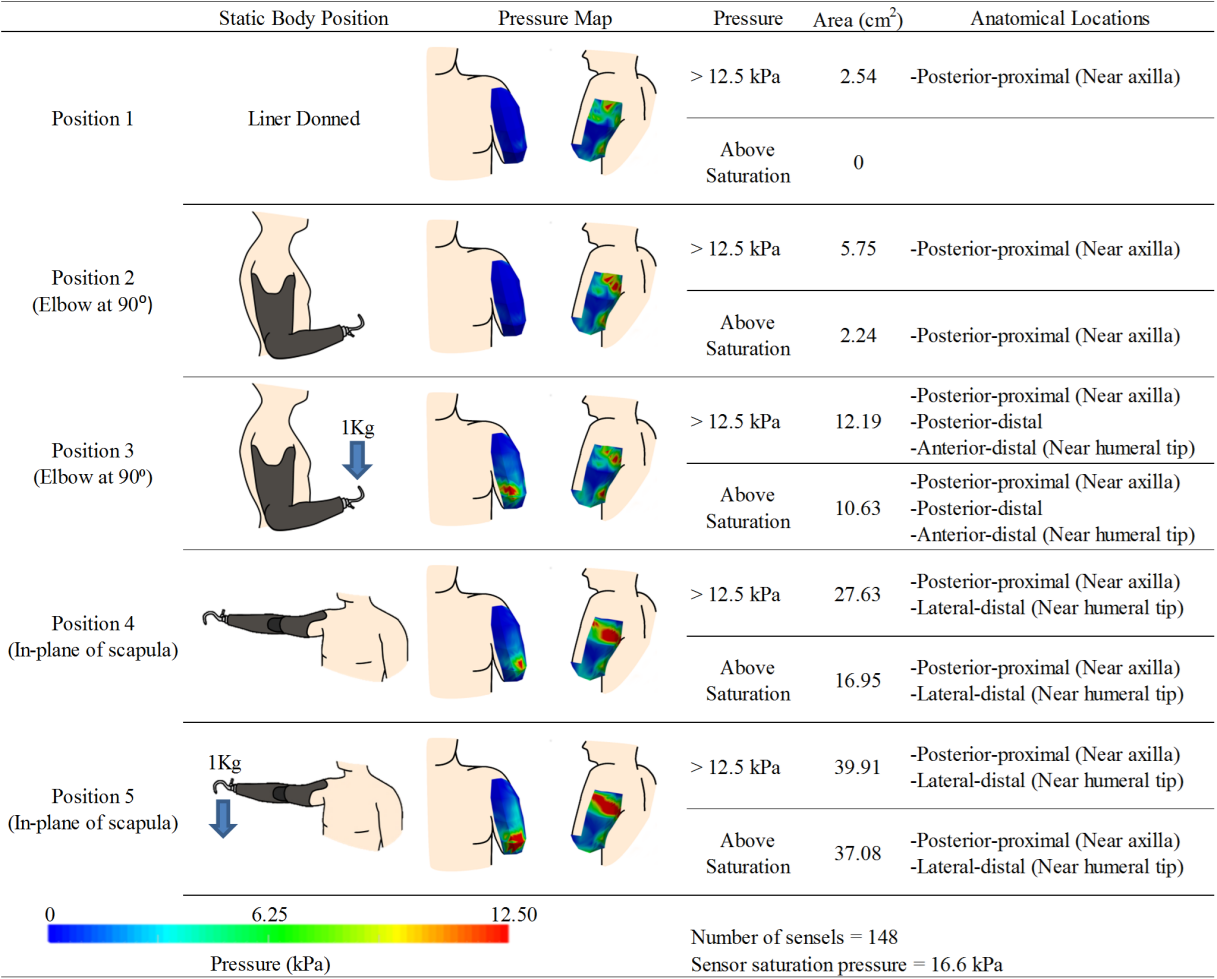
Experimental results for participant 1. kPa denotes units of pressure in kilopascals. Note: The posterior pressure maps are shown with the view in slight rotation to reveal pressures around the curve of the posterior axilla.

**Fig 4:**
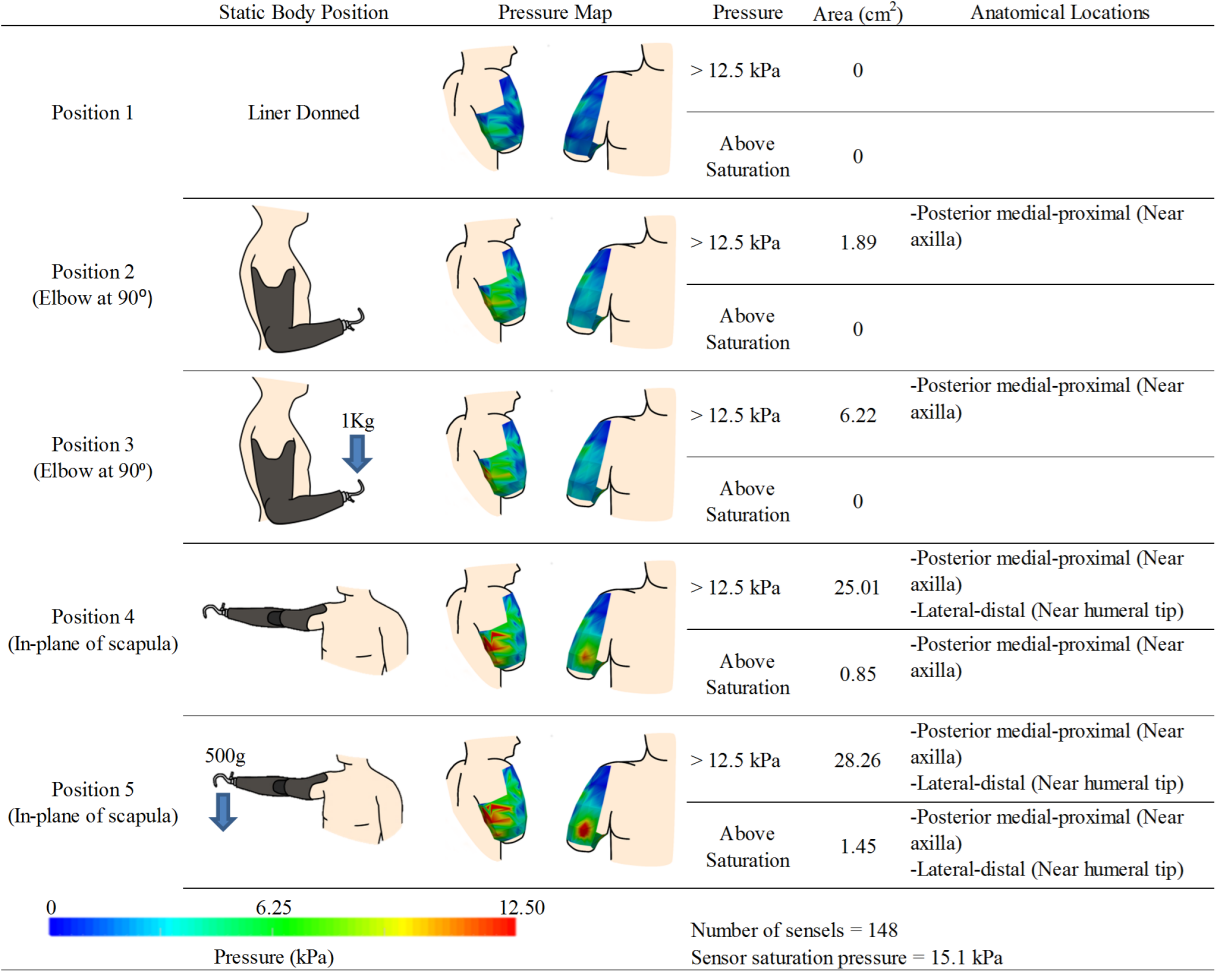
Experimental results for participant 2. kPa denotes units of pressure in kilopascals. Note: The posterior pressure maps view are shown with slight medial rotation to reveal pressures around curve of the posterior axilla.

**Fig 5:**
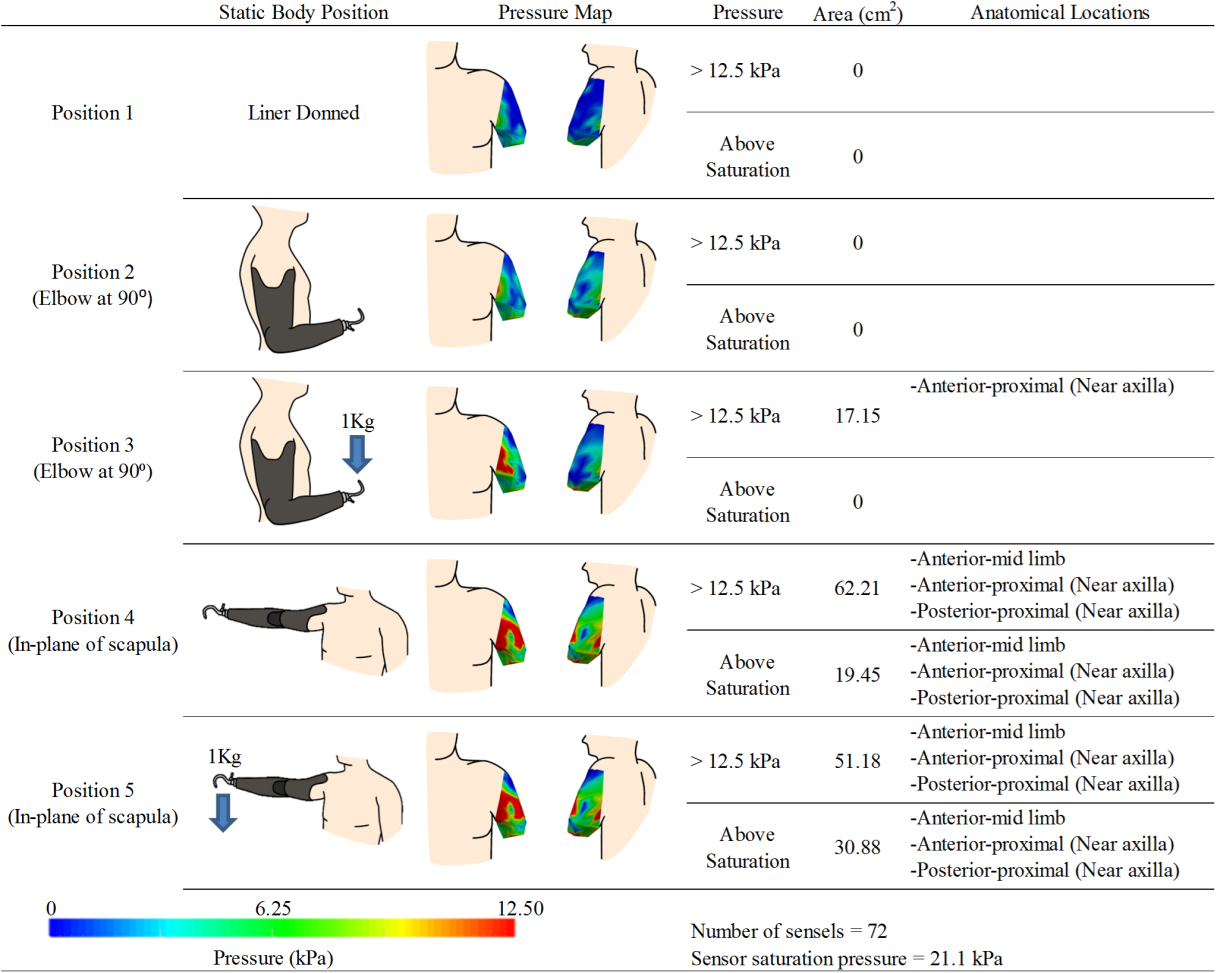
Experimental results for participant 3. kPa denotes units of pressure in kilopascals. Note: The posterior pressure maps view are shown with slight medial rotation to reveal pressures around curve of the posterior axilla.

**Fig 6:**
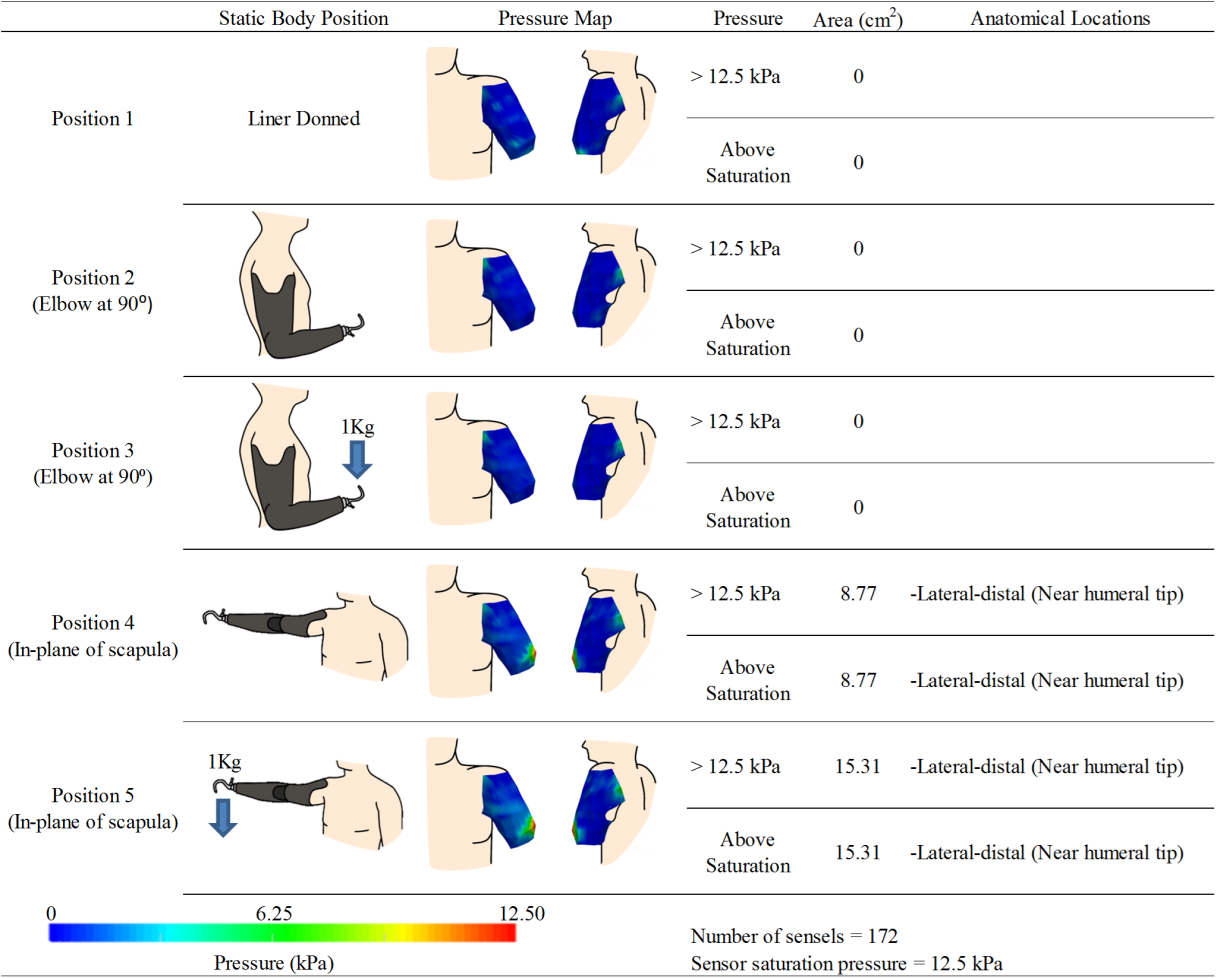
Experimental results for participant 4. kPa denotes units of pressure in kilopascals. Note: The posterior pressure maps view are shown with slight medial rotation to reveal pressures around curve of the posterior axilla.

Pressure measurements were then performed. Participants first donned their prosthetic liner, and were instructed to “hold still” with their RL positioned neutral at their side. The pre-pressure introduced by the liner was recorded. Participants then donned their prosthesis and pressure measurements were recorded in four static positions: 90° prosthetic elbow flexion; 90° prosthetic elbow flexion with 1 kg weight at their terminal device; full prosthetic elbow extension with shoulder flexion in the plane of the scapula; and, full prosthetic elbow extension with shoulder flexion in the plane of the scapula with 1 kg weight at their terminal device (Depicted in in Figs 3–6). Note that the prosthetic elbow was locked in each position indicated. Participants held each position statically for 3 seconds while pressure data was logged at 50 Hz.

### 2.3 Data treatment

For each participant performing a static pose, 150 samples of pressure data were recorded for each sensel (3 seconds at 50 Hz). Each sensel’s data (from the Tekscan system) was averaged and paired with its corresponding geometric location data (from the FARO arm system). This data set was then interpolated linearly to add two additional pressure values and corresponding coordinates between each sensel in preparation for creating surface pressure maps. Data was then imported into Paraview 5.0 software (Kitware Inc., Clifton Park, USA), where the ‘3D Delaunay Surface’ filter was employed to reconstruct the 3D geometry of each participant’s residual limb with overlaid pressure values. Pressure maps were scaled from 0 to 12.5 kPa, which was the lowest sensor saturation value of all the participants following sensor calibration; this value allows for a common scale for comparison of pressure maps across participants.

Threshold filtering was employed to isolate regions of maximum pressure, and the ‘Integrate Variables’ filter in Paraview was employed to estimate the corresponding surface areas of these regions on the RL. For each trial, the surface areas exceeding 12.5 kPa and exceeding the saturation pressure of each participant’s sensors were calculated.

## 3. Results

### 3.1 Participant 1

Participant 1 had a left side transhumeral amputation (the tested limb) and right side transradial amputation. It was approximately 11 years since the injury at the time of testing and the participant wore a transhumeral prosthesis between 12 to 15 hours a day. It was noted that participant 1’s RL was more abundant in soft tissue at the end of the RL, with less muscle tone relative to the other participants. Investigators noted redness and minor skin irritation near the posterior-proximal areas of their limb close to the axilla. However, the participant’s prosthetist evaluated the socket as being well-fit and the participant confirmed their satisfaction with the modified OPUS survey (S1 Table).

Two pressure sensors were used to cover the residual limb resulting in 148 sensels capturing discrete pressure measurements. Pressure maps and analysis results are highlighted in Fig 3. After calibration activities, sensor saturation occurred at 16.6 kPa. With the liner donned (Fig 3- Position 1) no area on the RL exceeded this threshold value. There was an area of higher pressure on the proximal posterior-medial aspect of the RL (near the posterior axilla) that may be an indication of the soft tissue contacting the thorax in this area. With the prosthesis donned and the elbow positioned at 90°(Fig 3- Position 2), the posterior-proximal region near the axilla registered the highest pressure. Surface areas on the RL exceeding 12.5 kPa were approximately 575 mm^2^ and those exceeding sensor saturation (16.6 kPa) were approximately 225 mm^2^. With a 1 kg load added to the terminal device (Fig 3- Position 3), the surface areas indicating more than 12.5 kPa or saturation increased to 1220 mm^2^ and 1065 mm^2^, respectively. Locations developing local pressure maximums included the posterior-proximal region near the axilla, the posterior-distal RL, and an anterior-distal region near the humeral tip. With the shoulder in flexion (Fig 3- Position 4), a large posterior-proximal region near the axilla and a smaller lateral-distal region near the humeral tip demonstrated local pressure maximums. The surface areas of the RL that exceeded 12.5 kPa or saturation were 2765 mm^2^ and 1695 mm^2^ respectively. This loading pattern was further amplified with the addition of a 1 kg load to the terminal device with the shoulder flexed (Fig 3- Position 5). Both local maximums grew in size with the areas that exceeded 12.5 kPa or saturation pressure were now 3990 mm^2^ and 3710 mm^2^ respectively.

### 3.2 Participant 2

Participant 2 had a right transhumeral amputation approximately 1 year prior to testing, and wore their prosthesis infrequently during the course of a day. The participant and their prosthetist confirmed that this was a well-fit socket; however, they noted that the prosthetic harness was “too tight” and caused irritation. However, above average device satisfaction was indicated by the modified OPUS survey (S1 Table).

Two pressures sensors were used to cover the residual limb resulting in 148 sensels capturing discrete pressure measurements. Pressure maps and analysis results are highlighted in Fig 4. After calibration, sensor saturation occurred at 15.1 kPa. With the liner donned (Fig 4- Position 1), no area on the RL exceeded 12.5 kPa. When the prosthesis was worn with elbow positioned at 90°(Fig 4- Position 2), a small area of 190 mm^2^ in the posterior medial-proximal area of the RL (near the axilla) exceeded 12.5 kPa, but did not exceed sensor saturation values (15.1kPa). This area increased to 625 mm^2^ with the addition of a 1 kg load on the terminal device (Fig 4- Position 3). Similar to Participant 1, with shoulder flexion (Fig 4- Position 4), posterior-medial regions of the RL increased in pressure. Additionally more of the posterior RL showed increased pressures, and a small area near the anterior humeral tip presented as a local pressure maximum. Surface areas exceeding 12.5 kPa or sensor saturation were 2500 mm^2^ and 195 3m^2^ respectively. Participant 1 was uncomfortable lifting a 1 kg load in abduction; therefore, a 500 g load was substituted (Fig 4- Position 5). This resulted in the areas that exceeded 12.5 kPa or saturation pressure growing to 2825 mm^2^ and 145 mm^2^ respectively.

### 3.3 Participant 3

Participant 3 had a left transhumeral amputation approximately 1 year prior to testing, and wore their prosthesis between 1 to 2 hours a day. Their prosthetist noted that special consideration was given during socket fabrication to accommodate sensitivity to pressure on the distal RL. During testing, Participant 3 noted that their harness may require readjustment as it was too tight and limited their range of motion. However, above average satisfaction was indicated upon completion of the modified OPUS survey (S1 Table).

Due to the relatively small size of Participant 3’s RL, one pressure sensor was trimmed and adhered to the limb resulting in 72 sensels capturing discrete pressure measurements. Pressure maps and analysis results are highlighted in Fig 5. After calibration, sensor saturation occurred at 21.1 kPa. With the prosthetic liner donned (Fig 5- Position 1), no area of the RL limb exceeded 12.5 kPa. Similarly, while the prosthesis was donned with the elbow at 90° (Fig 5- Position 2), no area of the RL exceeded 12.5 kPa, however a local maximum developed on the anterior-proximal side of the RL near the axilla. With the addition of a 1 kg load at the terminal device (Fig 5- Position 3), this local maximum increased to a 1715 mm^2^ area that exceeded 12.5 kPa, but no other area exceeded the sensor saturation value. When the prosthesis was held abducted (Fig 5- Position 4) the same anterior proximal area, the middle portions of the anterior RL, as well as the posterior-proximal area near the axilla demonstrated local pressure maximums. The areas that exceeded 12.5 kPa or saturation pressure were 6220 mm^2^ and 1945 mm^2^ respectively. When a 1 kg load was added to the terminal device (Fig 5- Position 5) the area that exceeded 12.5 kPa was reduced to 5120 mm^2^ while the area that exceeded saturation pressure grew to 3090 mm^2^.

### 3.4 Participant 4

Participant 4 had a left transhumeral amputation. It was approximately 10 years since their injury at the time of testing and they wore their prosthesis between 6 to 10 hours a day. Above average prosthetic satisfaction was indicated through the results of the modified OPUS survey (S1 Table).

Two pressures sensors were used to cover the residual limb resulting in 172 sensels capturing discrete pressure measurements. Pressure maps and analysis results are highlighted in Fig 6. After calibration, sensor saturation occurred at 12.5 kPa. With the liner donned (Fig 6- Position 1) no area of the RL limb exceeded 12.5 kPa. With the prosthesis donned and elbow at 90°, both with and without the 1 kg load, no area of the limb exceeded 12.5 kPa (Fig 6- Position 2 and 3). In these positions a local maximum (although relatively low in magnitude) was observed in the posterior-proximal area of the RL near the axilla as well as anterior proximal region. With the shoulder flexion (Fig 6- Position 4), a local pressure maximum was observed on the lateral-distal region of the limb near the humeral tip with an approximate surface area of 875 mm^2^. This area expanded to 1530 mm^2^ with the introduction of a 1 kg load at the terminal device (Fig 6- Position 5).

## 4. Discussion

One of the most influential factors for the use of upper limb prostheses is the socket, which must be custom designed to accommodate the individual’s morphology and to distribute the pressures resulting from the weight of the prosthesis appropriately across the RL. Socket fabrication currently relies heavily on heuristic practice. This work presents a first step toward incorporating a quantitative-empirical tool into UL prosthetic socket evaluation. In a fabrication context, one of the most useful methods presented in this work is the ability to characterize pressure distribution patterns and identify anatomical locations bearing local pressure maximums, to further understanding of what constitutes a “well-fit” socket.

For each participant, individual socket design considerations were reflected in the surface pressure distribution across their RLs. Proximal areas of the RL near the axilla were a common area of high pressure in Participants 1, 2, and 3. During socket fabrication, this area was specifically targeted by the prosthetist for tissue compression as a method of avoiding socket contact with the user’s thorax. This technique allows increased mobility of the shoulder joint and the additional tissue compression helps stabilize the socket in the coronal plane; however, as a result, it leads to higher pressures while the prosthesis is worn and a possible destabilizing effect in the sagittal plane. Investigators noted mild tissue irritation in this area on Participant 1. Being a user who has undergone bilateral amputation, their dependency on the prosthesis is greatly increased, which is reflected in typical daily use (12–15 hours/day). Therefore, the increased pressure in this area coupled with high usage time, and the noted abundance of soft tissue, likely increases the risk of tissue irritation. However, it was also noted that Participant 1 reported no discomfort while wearing the device and confirmed that the socket was in fact well-fit.

Participant 3’s socket was unique relative to the other participant’s as it was fabricated to accommodate sensitivity of the distal RL. The prosthetist specifically targeted middle regions of the anterior RL for tissue compression, thereby relieving distal load bearing. This is evident in the location of local pressure maximums in the resulting pressure maps. When the prosthesis was positioned with the shoulder in an abducted position, an anterior region at the mid-length of the limb, as well as a posterior area near the axilla, exhibited local pressure maximums. When a 1 kg load was added in this position, the surface area exceeding saturation pressure (21.2kPa) increased, yet the area exceeding 12.5kPa decreased. This suggests that with further loading of the terminal device, the socket continued to concentrate its application of pressure over the high pressure areas rather than further redistributing the load, thereby successfully avoiding excess pressure onto the distal limb.

Tissue deformation around skeletal structures was evident in the pressure maps of Participants 1, 2, and 4; especially with the prosthetic elbow and participant’s shoulder extended. Local pressure maximums near the distal tip of the residual humerus were observed, with the formation of high pressure areas posterior-medially on the RL in Participants 1 and 2. These patterns were further intensified and grew in surface area with a load applied to the terminal device. In this position, the prosthesis’ center of gravity is distal-lateral to the socket. This results in a fulcrum by which the rigid prosthetic socket pivots about the humeral tip which is counter balanced by the posterior medial regions of the RL. In more extreme cases, this action may result in lateral gapping, whereby a void is created as the socket loses contact with the RL in some areas while concentrating pressure on opposing locations. This phenomenon can increase discomfort, and reduce the desire to use the device in greater ranges of motion. Less severe forms of this loading pattern are nearly unavoidable due to the physics of encapsulating soft deformable tissue between a rigid socket and skeletal structure. Some novel socket designs attempt to minimize lateral gapping effects through strategic patterns of tissue compression and release[7], with follow up imaging and user satisfaction studies being conducted to evaluate the efficacy of their design[24]. The analytical techniques presented in this work would enable the evaluation of the tissue loading mechanism proposed in these novel socket designs, through the identification of the affected anatomical areas as well as a description of the size and relative magnitude of the pressure in these areas.

The techniques presented in this work demonstrate a number of encouraging potential benefits if incorporated for socket evaluation. Implemented clinically, these techniques can help the prosthetist further refine socket geometry by specifically and accurately targeting regions of a patient’s anatomy for load bearing. Beyond this, the data also highlights anatomical areas bearing little or no load. As prosthetic weight, internal socket temperature, and resulting perspiration are often highlighted as major contributors to discomfort, tissue damage, and prosthetic abandonment[25], there exists the possibility that the corresponding non-loaded areas of the socket may be removed. This may help reduce some the undesirable effects of wearing a traditional prosthetic socket, by providing a lighter more breathable device that maintains the interfacial mechanics necessary for suspension and control.

As novel sockets continue to be developed for improved comfort, stability and suspension, this work presents a tool that can be implemented to analytically evaluate new approaches. By quantitatively documenting socket designs to understand the effect on the underlying interfacial mechanics, UL socket fabrication might begin to be more approachable by those less experienced in the highly specialized techniques. However, adoption into a clinical setting will require further development with regards to ease of use and technical accessibility. The Tekscan VersaTek system used to capture contact pressure data is commercially available and designed specifically for the clinic. The procedures presented for capturing RL geometry could be modified to use commercially available laser scanners that are already present in many prosthetic workshops and computer aided design (CAD) systems. However, a gap currently exists in the technical knowledge required to easily pair this data and assemble pressure maps relative to the anatomy. Therefore the development of automated software capable of quickly and accurately assembling pressure maps, with reduced requirement for technical expertise, will be necessary to promote clinical adoption.

Upper limb socket design and fabrication is a process that must accommodate and optimize numerous considerations specific to a patient’s morphology, anatomy, and comfort, among many other factors. This work highlights four participants, all with clinically deemed “well-fit” prosthetic sockets, who demonstrated similar and unique load bearing characteristics at the interface of their RL and socket. Although the results were individual to each participant, the presented techniques provided a quantitative understanding of the implications of some of the major design decisions made during socket fabrication. Therefore, this work can help provide a foundation for techniques aimed at leveraging analytically-based design practices, by furthering our understanding of transhumeral socket interface mechanics.

### 4.1 Limitations

A well-documented limitation of Tekscan measurement systems is the measurement accuracy. The Tekscan VersaTek system and 9811E sensor used in our study have been reported to have measurement error of 8.5% on flat surfaces and 11.2% on curved surfaces[16]. To help mitigate sensor error, it has been suggested that calibration activities be performed in an environment as close to its intended use as possible[23] (in this case directly affixed to the RL). In doing so, the sensors must be bent to conform to the RL prior to calibration. The physical bending of the sensors introduces an unavoidable initial change in sensel resistance that reduces their working range and results in earlier sensor saturation. Therefore, the technique used in our approach allows a more accurate representation of how pressure distributes across the RL and identification of maximum pressure locations. However, we are unable to infer the magnitude of the pressure in the localized areas of sensor saturation.

One or two sensors were placed on each participant’s RL. The investigator attempted to maximize coverage; however full coverage of the limb was not physically possible. Although we could discriminate between 72 to 172 discrete pressure readings (depending on the participant), values occurring in-between sensors and sensels are still unknown. Future work with more densely packed sensor arrays and sensors more adaptable to RL geometry could help improve this resolution.

Finally, two body positions representing a relatively neutral position (arm at the side) and the maximum range of motion for the shoulder were performed by participants under two loading conditions. However, this does not represent the full range of uses for transhumeral prostheses. Although this work provides a preliminary understanding of RL-socket interaction in relation to body position, further work with dynamic movements or other functional static body positions may be warranted.

## Supporting information

S1 Table Modified OPUS survey results. Note: 50th percentile (average) results in the Satisfaction with Device Survey has a Measure of approximately 45 and Score of 22.
(DOCX)
