# Supplementary material for: Characterization of interfacial socket pressure in transhumeral prostheses: A case series
Source: PLoS One. 2017 Jun 2;12(6):e0178517. doi: 10.1371/journal.pone.0178517 (PMC5456071; doi:10.1371/journal.pone.0178517)
Supplement: S1 Table — Note: 50th percentile (average) results in the Satisfaction with Device Survey has a Measure of approximately 45 and Score of 22. (DOCX) [file pone.0178517.s001.docx]

**S1 Table**: Modified OPUS survey results. Note: 50^th^ percentile (average) results in the *Satisfaction with Device Survey* has a Measure of approximately 45 and Score of 22
